# South Asian individuals with diabetes who are referred for MODY testing in the UK have a lower mutation pick-up rate than white European people

**DOI:** 10.1007/s00125-016-4056-7

**Published:** 2016-07-19

**Authors:** Shivani Misra, Beverley Shields, Kevin Colclough, Desmond G Johnston, Nick S Oliver, Sian Ellard, Andrew T Hattersley

**Affiliations:** 1 Diabetes, Endocrinology and Metabolism, Imperial College London, Ground Floor Medical School Building, St Mary’s Campus, Norfolk Place, London, W2 1PG UK; 2 Institute of Biomedical and Clinical Science, University of Exeter Medical School, Exeter, UK; 3 Department of Molecular Genetics, Royal Devon and Exeter NHS Foundation Trust, Exeter, UK

**Keywords:** Glucokinase, Hepatic nuclear factor 1 alpha, Hepatic nuclear factor 4 alpha, MODY, South Asian

*To the Editor*: Maturity onset diabetes of the young (MODY) is an autosomal dominantly inherited form of diabetes that has been extensively described in white populations, but diagnosed with lower frequency in other ethnic groups. Mutations in the *GCK*, *HNF1A* and *HNF4A* MODY genes have been reported in a variety of ethnic groups [1–3], but there are few data on prevalence in non-white ethnic groups. MODY was first reported in a UK South Asian (SA) cohort in a systematic survey of childhood diabetes [4]. A 2006 study revealed a lower than expected frequency of referrals for MODY testing in the UK SA population [5]. The frequency and characteristics of MODY in non-white European populations is important for the precise diagnosis of young-onset diabetes.

We undertook a retrospective analysis of UK referrals for MODY testing to determine if referrals for individuals of SA origin had increased since that reported in the 2006 study and to investigate the pick-up rate and clinical features of MODY mutations in this population.

The UK MODY database, held centrally at the Royal Devon and Exeter National Health Service (NHS) Foundation Trust, is populated prospectively from a standardised request proforma that includes ethnicity. Proband referrals (>1 year old at diagnosis) from 1996 to November 2015, in whom sequencing for mutations in the most common MODY genes (*HNF1A*, *HNF4A*, *HNF1B* or *GCK*) was requested, were selected. Only variants classified as pathogenic or likely pathogenic were included. Assignment of pathogenicity was based on UK best practice guidelines [6]; including findings from established variant/mutation database and literature searches, details of familial cosegregation and identification of clinical features characteristic of the MODY gene mutation. Additionally, data from in silico prediction models (SIFT, PolyPhen2, AlignGVGD, Grantham distance accessed using AlaMut Visual 2.7, Interactive Biosoftware, Rouen, France) were used, along with details of amino acid species conservation and predictions of effects on splicing. Finally, variants in the Exome Aggregation Consortium [7] and 1000 genomes [8] databases with a minor allele frequency >0.01, were excluded.

Ethnicity was categorised as white European (WE) or SA based on UK census classification [9]. The SA group included people from India, Pakistan and Bangladesh or in whom ethnic origin was stated to be ‘Asian’ and excluded mixed ethnicity.

A total of 4688 proband referrals were identified and ethnicity was recorded in 4010 cases. White European referrals accounted for 3472 of 4010 (86.6%). 293 cases referred were in people of SA ethnicity (7.3%), and 81 referrals (2.0%) were from African or Caribbean backgrounds (see Table 1).

**Table 1:** Breakdown of proband referrals for MODY genetic testing and MODY pick-up rate by ethnic group

Ethnic group	*n*	%	MODY mutation (*n*)	Pick-up rate (%)
All groups	4688		1180	25.2
Individuals with known ethnicity	4010		1083	27.0
White	3472	86.6	1011	29.1
South Asian	293	7.3	37	12.6***
African-Caribbean	81	2	8	9.9
Middle Eastern	47	1.2	5	10.6
South East Asian	21	0.5	6	28.6
Mixed/other	96	2.4	16	16.7
All non-white ethnic groups	538	13.4	72	13.4

Statistical analysis was carried out using a *χ* ^2^ test on the two largest ethnic groups, white and South Asian

***p < 0.001 vs white individuals

1180 probands had MODY mutations, with a mutation pick-up rate of 25.2% (1180/4688). The mutation pick-up rate was 29.1% (1011/3472) in the WE group and 12.6% (37/293) in the SA group (*p* < 0.001) (see Table 1). The pick-up rate in children (<12 years) referred for testing did not differ between ethnic groups; 32.6% in WE vs 26.7% in SA children, *p* = 0.356 (data not shown).

Phenotypes of those with and without confirmed MODY mutations are displayed in Table 2. Generally, SA MODY patients with *GCK* mutations were diagnosed earlier than WE patients (6.5 vs 20 years, *p* < 0.0001); however, when only considering diagnosis in children under 12 years old, there was no significant difference in age at diagnosis. Referrals from both ethnic groups had similar proportions of a history of parental diabetes and were equally likely to be non-insulin dependent.

**Table 2:** Clinical characteristics of referrals with *GCK* or *HNF1A* / *HNF4A* MODY mutations and those without MODY mutations, analysed by ethnic group

Mutation status	No mutation			*GCK*			*HNF1A* / *HNF*4A		
Variable	WE	SA	*p* value	WE	SA	*p* value	WE	SA	*p* value
Total (*n*)	2387	256		358	17		545	15	
Age at diagnosis (years)	25 (16–35)	19 (14–30)	<0.0001***	20 (14–28)	6.5 (4.5–15)	<0.0001***	18.5 (15–25)	16 (14–20)	0.054
Age at diagnosis in children ≤12 years (years)	9 (6–11)	11 (7–11.5)	0.012*	8 (5–10)	5 (3–7)	0.079	11 (9–12)	11 (8–12)	1.000
Current age (years)	35 (25–47)	28 (19–36)	<0.0001***	29 (19–39)	13 (6–17)	0.0044**	37 (26–48)	25 (20–48)	0.108
Duration of diabetes (years)	7 (2–14)	4 (2–9)	<0.0001***	6 (2–11)	2 (0–5)	0.0045**	14 (6–27)	11 (6–28)	0.658
BMI (kg/m^2^) in patients aged ≥16 years	25.3 (22.4–29.6)	25.0 (23.0–28.2)	0.217	22.1 (20.1–25.0)	19.7 (18.0–20.3)	0.02*	24.1 (22.0–27.0)	24.0 (21.1–24.6)	0.246
Fasting glucose (mmol/l)	7.4 (6.1–10.1)	7.9 (6.5–10.7)	0.223	6.7 (6.3–7.3)	6.9 (6.5–7.1)	0.918	7.8 (6.3–9.9)	7.3 (6.7–8.2)	0.638
HbA_1c_ (%)	7.4 (6.3–9.1)	7.8 (6.7–9.7)	0.0065**	6.4 (6.1–6.7)	6.6 (6.5–6.9)	0.04*	7.2 (6.3–8.4)	8.6 (8.1–8.8)	0.004**
HbA_1c_ (mmol/mol)	57 (45–76)	62 (50–83)	0.0065**	46 (43–50)	49 (48–52)	0.04*	55 (45–68)	70 (65–73)	0.004**
Meets all three clinical criteria (%)^a^	14.8	31.3	<0.0001***	43.5	85.7	0.002**	44.7	50	0.736
Diagnosed <25 years (%)	43.4	58.6	<0.0001***	61.5	94.1	0.006**	72.1	93.3	0.069
Currently non-insulin treated (%)	50.6	60.2	0.006**	89.7	92.3	0.762	65.7	69.2	0.794
History of parental diabetes (%)	68.2	83.6	<0.0001***	83.5	93.8	0.274	89.9	92.9	0.720

Data are presented as medians and interquartile ranges or %

^a^Calculated using cases where the data set for all parameters is complete

Statistical analysis was carried out using Mann-Whitney U / *χ* ^2^ tests

*p < 0.05, **p < 0.01, ***p < 0.001

In both SA and WE individuals, the *HNF1A / HNF4A* MODY phenotypes were similar across all variables reported except for HbA_1c_, which was higher in the SA group (8.6% [70 mmol/mol] vs 7.2% [55 mmol/mol], *p* = 0.004; see Table 2).

SA people in whom no MODY mutations were found were younger at diagnosis (19 years vs 25 years, *p* < 0.0001) and had a shorter duration of diabetes, compared with WE individuals. BMI was similar across both ethnic groups (WE, 25.3 vs SA 25.0 kg/m^2^, *p* = 0.217). However, SA people were more likely to have a parent affected with diabetes (83.6% vs 68.2%, *p* < 0.0001) and were more likely not to be treated with insulin at referral (60.2% vs 50.6%, *p* = 0.006), when compared with WE individuals (see Table 2).

This study is the first to explore the characteristics of individuals of SA ethnicity who have been referred for MODY testing using data from the largest MODY diagnostic service in the UK. It demonstrates a lower pick-up rate of MODY mutations in people of SA ethnicity. In our analysis, SA people accounted for markedly more referrals than previously reported in 2006 (7.3% vs 0.5%) [5]. Whilst this broadly reflects the UK population demographics, because of the higher prevalence of type 2 diabetes in SA people it is suggested that this is still lower than expected [9]. The detection rate for MODY mutations was lower in the SA group than in WE referrals. However, when a mutation was not detected, SA referrals were more likely to meet the clinical criteria for MODY than WE individuals. This may demonstrate a referral bias favouring SA cases that more stringently meet the clinical criteria. However, the results also show that these criteria are not sensitive for the separation of MODY from young-onset type 2 diabetes in this population. For example, SA people without mutations had higher BMIs than those with confirmed MODY mutations, suggesting that the low MODY detection rate in this group may have resulted from a higher prevalence of young-onset, familial diabetes.

When analysing the SA group as a whole, the best predictors differentiating those with MODY (mutations in all genes) from those without a mutation, were a lower BMI and age at onset; BMI was lower in the SA group with MODY mutations than those without mutations (MODY, 21.4 kg/m^2^ and no MODY, 25.0 kg/m^2^, *p* = 0.001, data not shown), but BMI was generally higher and non-significantly different in the no mutation group when compared with WE people (25.0 vs 25.3 kg/m^2^, respectively). The lower BMI and age at diagnosis in SA referrals was primarily observed in the *GCK* mutation group and not seen in the *HNF1A / HNF4A* mutation or ‘no mutation’ groups. It is likely that these findings reflect a lower referral rate in adults and children over 12 years old since the mutation pick-up rate in both ethnic groups was similar in children under 12 years.

There is limited evidence to support that SA individuals have a lower prevalence of MODY, with only one previous study suggesting fewer *GCK* mutations occurred in Indian children [10]; however, this study may have been underpowered since the estimated minimum prevalence of MODY (in a predominantly white population) has recently been demonstrated to be 1 in 1000 cases [11]. Thus, it is more likely that the lower mutation pick-up rate in SA individuals compared to WE individuals observed in our study reflects higher prevalence of younger-onset type 2 diabetes, which is supported by our analysis demonstrating similar mutation pick-up rates between ethnic groups in pre-pubertal children.

Our data highlight the need to consider the optimum method for identifying cases of MODY in people of non-white European ethnicity. Approaches such as the MODY probability calculator [12] need to be validated in non-white populations as our data suggest that MODY cases may be more sensitively identified using lower cut-offs for age at diagnosis and BMI.

Referrals for MODY testing in the SA population have increased considerably but may still be under-representative of the general UK population. Meanwhile, despite meeting referral criteria more stringently, the lower MODY mutation pick-up rate for SA individuals suggests that these criteria are not sensitive for separating MODY from younger-onset type 2 diabetes in the UK SA population.

Further work is required in unselected cohorts, to develop ethnic-specific criteria that facilitate the identification of MODY from type 2 diabetes in SA people with young-onset diabetes.

## Abbreviations

SA: South Asian
WE: White European
